# A Linguistic Hierarchy Model with Self-Confidence Preference Relations and Its Application in Co-Regulation of Food Safety in China

**DOI:** 10.3390/ijerph16162918

**Published:** 2019-08-14

**Authors:** Sha Fan, Hengjie Zhang, Huali Tang

**Affiliations:** 1 Business School, Sichuan University, Chengdu 610065, China; 2 Business School, Hohai University, Nanjing 211100, China

**Keywords:** linguistic preference relations, self-confidence, nonlinear programming model, hierarchical structure, group decision making, co-regulation of food safety

## Abstract

Linguistic preference relations are widely used by decision makers to elicit their preferences over alternatives in the Group Decision Making (GDM) process. Recent studies have shown that self-confidence, as an important human psychological behavior, has an important influence on decision-making results. However, multiple self-confidence levels of decision makers are seldom considered in the linguistic preference relation. Meanwhile many real-word decision-making problems are analyzed in a hierarchical structure, in which a complicated problem can be divided into several easier comprehended sub-problems. Hence, this paper aims at designing a linguistic hierarchy model with self-confidence preference relation (LHM-SCPR) to discuss complex GDM problems in a hierarchical structure. In the SC-LPR, each element contains two components, the first one is the preference value between pairs of alternatives, and the second one that is defined on a linguistic term set represents decision maker’s self-confidence level associated to the first component. Meanwhile, a nonlinear programming model is proposed to derive individual preference vector from SC-LPR. Then, we apply LHM-SCPR in co-regulation of food safety to present the validity of this method, and find that improving the participation skills regarding co-regulation of food safety is the most pressing task. Finally, detailed comparative analysis and discussion are presented to verify the validity of the proposal.

## 1. Introduction

Group decision making (GDM) can be defined as an activity in which two or more decision makers differ in preference, recognize regarding a common problem and attempt to reach a collective decision [1]. Hierarchical structure, as a useful tool to analyze complicated GDM problems, was firstly proposed by Saaty [2], and also is utilized by decision makers to divide a complex decision making (DM) problems into hierarchal simpler sub-problems [2], so hierarchical structures have been wildly used in many applications related with DM problems, such as risk management [3], supplier selection and evaluation [4], and so on.

In GDM processes, preference relations are utilized by decision makers to express their preference information [5,6,7,8]. There are several types of preference relations, such as multiplicative preference relation [2], additive preference relation [9] and linguistic preference relation [10]. Among above preference relations, linguistic preference relation is widely used in day-to-day decision-making activities because that it facilitates decision makers’ elicitation of linguistic evaluations. Decision makers are likely to show multiple self-confidence levels in their preference information due to the difference of their knowledge and experience. Self-confidence has an important influence on decision making [11], which has received several researchers’ attention recently. Liu et al. [5] introduced a new type of preference relation called linguistic preference relation with self-confidence (SC-LPR), which allows decision makers to provide their preference information with multiple self-confidence levels. Two components are composed in SC-LPR, the preference value between pairs of alternative, and the self-confidence level associated to the first component.

Hierarchical structure has been widely used to divide complex decision-making (DM) problems into hierarchy simpler sub-problems in extant literatures. SC-LPR allows the decision maker to express preference information with multiple self-confidence levels, so this paper aims to propose a novel method for handling complex GDM problems: linguistic hierarchy model with self-confidence preference relation (LHM-SCPR). Steps of LHM-SCPR can be briefly summarized as following: (1) build a hierarchical structure model regarding a GDM problem; (2) a group of decision makers provide SC-LPRs; (3) check the consistency of SC-LPRs; (4) a optimization model is developed to gain individual preference vector via a nonlinear programming model; (5) obtain a collective preference vector by aggregating individual preference vectors; (6) synthesize collective preference vectors to produce the global priority vector regarding alternatives.

According to WHO statistics, there are approximately two million fatal case of food poisoning every year around the world. Moreover, huge disease burden from food highlights the importance of food safety, particularly in developing countries, such as Africa, Asia. Food safety is critical in food production and food processing, which are categorized as agriculture food safety and industry food safety [12]. According to data from the National Bureau of Statistics of China, we know that China is an agricultural country. For example, in 2017, the total agricultural output value accounted for 53% of the total primary industry output value. The transformation between a large-scale agricultural production economy and a food industrial economy involves agricultural food safety and industry food safety in the long chains of food production and food processing. Moreover, food safety issues are likely to occur at any part of these chains. What’s more, if food safety issues disseminate and keep simmering on the internet, panic and disorder may be caused among society.

Although the Chinese government has done a lot of work on food safety in recent years, such as issuing the Food Safety Law of the People’s Republic of China, strengthening the food safety control system via the Food Safety Risk Surveillance System, Food Safety Risk Assessment System and Food Safety Standards System, major food safety accidents still happen sometimes. Table 1presents some major food safety incidents that have caused adverse influence on our society in recent years. Obviously, the food safety problem has not been fundamentally resolved yet. In a word, food safety is still a hotspot that cannot be ignored in China.

Up to now, there are some studies regarding food safety governance. Unnevehr et al. [13] proposed that the public-private partnership can support the process of improving food safety management. Zhang et al. [14] conclude that media and consumer are the major players in the third-party regulation. Chen et al. [15] proposed that taking measure to train the public can improve the public abilities for taking part in co-regulation of China’s food safety. Liu et al. [16] proposed that the government should pay more attention to public perception of food safety and work with other entities, such as food industry and non-governmental organization (NGO). Through the review of these above literatures, we can know that it is urgent to motivate the public participate in co-regulation of food safety for improving governance effectiveness.

Food safety is a global problem, which is related to human beings’ health. Solving this problem needs the public and government sector to coordinate their respective regulatory activities. So, food safety can be discussed from the perspective of GDM. However, from the review of the above literatures, it is not difficult to find that discussing food safety governance from the perspective of GDM is still a challenge. To overcome the mentioned limitation, this paper will apply the proposed novel GDM method in co-regulation of food safety.

The rest of this paper is organized as follows: Section 2 reviews some literature related to MCDM techniques. Section 3 introduces the required background in GDM. Section 4 presents a novel framework for complex GDM: linguistic hierarchy model with self-confidence preference relations. In Section 5, the proposed method is applied in co-regulation of food safety, and then, a comparative analysis is presented. Section 6 presents some contributions and limitations of the proposed model are discussed. Lastly, conclusions are shown in Section 7.

## 2. Literature Review

This section reviews some related works regarding the multi-criteria decision making (MCDM) techniques that have been used for addressing GDM problems, as well as some preference relations that have been used by decision makers to provide their evaluation information. Based on the summary and analysis of many references, we will explain the reason why we choose the proposed model in our paper.

There are many references about multi-criteria decision making (MCDM). MCDM is a field of operations research that aims at developing and implementing decision support tools and methodologies to tackle with complex decision problems involving multiple criteria, goals, or objectives of conflicting nature [17]. 

Due to the different natures of MCDM problems, several methods have been developed, such as TOPSIS [18], preference ranking organization method for enrichment evaluation (PROMETEE) [19], elimination and choice expressing reality (ELECTRE) [20], analysis network process (ANP) and AHP [2,21], step wise weight assessment ratio analysis (SWARA) [22], decision making trial and evaluation laboratory (DEMATEL) [23], simple additive weighting (SAW) [24], and VIKOR [25]. The above MCDM methods have been widely used to address many real-world decision problems. For example, Micale et al. [26] combined ELECTRE and TOPSIS for solving the storage location assignment problem. Akgün et al. [27] combined AHP-TOPSIS and GIS to solve an ammunition distribution network design problem. DEMATEL and ANP were combined for evaluating the performance of hospital supply chain [28]. VIKOR method was applied in green supplier development program selection [29]. Sirisawat et al. [30] used fuzzy AHP-TOPSIS approaches to prioritizing solutions for reverse logistics. Ramkumar et al. [31,32,33,34] extended ANP and applied it in DM problems of e-procurement services. Sharma et al. [35] proposed Bayesian belief networks (BBN) based on the AHP and ANP for dynamic temporary blood facility location-allocation. Kim et al. [36] combined DEMATEL based on ANP and the complex proportional assessment of alternatives to grey relations (COPRAS-G) technique to select logistic service providers. Liu et al [37] proposed the ranking range based on MADM approach under incomplete context and applied it to venture investment evaluation, and so on. From the review of previous studies regarding MCDM methods, we can find that several kinds of MCDM methods and hybrid methods of MCDM are applied to handle decision problems under different situations.

Among the above MCDM methods, AHP is one of the most widespread and significant MCDM methods because of its efficiency in dealing with complex DM problems, especially these problems involving a large number of alternatives and multilevel criteria. AHP can be used to aggregate the priority for all level of hierarchy structure including the level representing alternatives, however, the other MCDM methods can’t implement this aggregate calculation.

Although AHP has been widely used for handling general decision making problems, it also has the limitation in the use of AHP for uncertain DM problems. For example, the traditional AHP described by Saaty’s 1–9 scale can’t be applied in the following situation that decision makers are likely not to provide their opinions with an accurate number with respect to the decision-making environments’ uncertainty and vagueness. In order to improve the practicability of AHP, some scholars have combined fuzzy set theory [38] (such as trapezoidal fuzzy numbers [38] and triangular fuzzy number [39]) with AHP. Then, some studies has been conducted to extend the DM method within the context of fuzzy AHP [30,40]. However, these fuzzy AHP approaches can only be used to deal with quantitative information [41], and sometimes, individual preference information regarding an alternative is often vague and can’t be expressed with a crisp number. It may be convenient for decision makers to provide their preference information regarding an alternative with qualitative linguistic description. As we all know, real-world GDM problems involve not only the mathematics but also human psychological behaviors. Self-confidence as one of human psychological behaviors has important impact on decision making. However, according to the above literatures, few of them introduce linguistic preference relations into traditional GDM method, and self-confidence is ignored in the process of decision making. Hence, this paper proposes a novel method for complex GDM problems by combined hierarchical structure and linguistic preference relations with self-confidence.

## 3. Preliminaries

In this section, preliminary concepts regarding the 2-tuple linguistic model and preference relations, linguistic preference relations with self-confidence and a hierarchical structure used in this framework are introduced.

### 3.1. The 2-Tuple Linguistic Model and Preference Relation

In linguistic decision-making problems, 2-tuple linguistic model is widely used for computing with words. In this paper, we use 2-tuple linguistic model to carry out computing with words when dealing with the linguistic self-confidence levels information. Herrera et al. [42] introduced the basic notations and operational laws of ordinal linguistic variables, a summary of which is provided below.

Let $S=\left\{s_{i}i=0,1\ldots g\right\}$ be a linguistic term set with odd cardinality. The term $s_{i}$ represents a possible value of linguistic variable. The ordinal ordering on set S is assumed: $s_{i}>s_{j}$ if and only if i> j. The 2-tuple linguistic model presented by Herrera et al. [42] is based on the Definition 1.

**Definition** **1.** (Herrera et al. [42]). *Let β∈[0,g] be a number in the granularity interval of the linguistic term set S $=\left\{s_{i}i=0,1\ldots g\right\}$ and let i=round(β) and α=β−i be two values such that i∈[0,g], and α∈[−0.5,0.5), where round is the usual rounding operation,”α” is the value of the symbolic translation.*

The 2-tuple linguistic model represents the linguistic information by means of 2-tuples $\left(s_{i},\alpha \right)$, where $s_{i}\in S$ and α∈[−0.5,0.5), which defines a one-to-one mapping function between linguistic 2-tuples and numerical values in [0,g].

**Definition** **2.** (Herrera et al. [42]). *Let $S=\left\{s_{0},s_{1},\ldots ,s_{g}\right\}$ be a linguistic term set and β∈[0,g] be a value representing the result of a symbolic aggregation operation. The 2-tuple that expresses the equivalent information to β is obtained with a one-to-one mapping function: Δ:[0,g]→S×[−0.5,0.5), where:*

$$\Delta \left(\beta \right)=\left(s_{i},\alpha \right),\mathrm{with}\{\begin{array}{c} s_{i},i=round\left(\beta \right)\\ \alpha =\beta -i,\alpha \in \left[-0.5,0.5\right) \end{array}$$

For convenience, we denote S* = S × [−0.5, 0.5). Accordingly, the inverse function of Δ is $\Delta^{-1}:$ S∗→[0,g] and $\Delta^{-1}\left(s_{i},\alpha \right)=i+\alpha =\beta .$ Additionally, an ordering on the set of 2-tuples and a negation operator are defined by Herrera et al. [42] and Dong et al. [43] as following:

(1) 2-tuples comparison operator: Let $\left(s_{k},\alpha \right)$ and $\left(s_{l},\gamma \right)$ be two 2-tuples, then:
  - if k>l, then $\left(s_{l},\gamma \right)$ is smaller than $\left(s_{k},\alpha \right)$;
  - if k=l, then
    (a) if α=γ, then $\left(s_{k},\alpha \right)$ and $\left(s_{l},\gamma \right)$ represents the same information;
    (b) if α<γ, then $\left(s_{k},\alpha \right)$ is smaller than $\left(s_{l},\gamma \right)$;
(2) A 2-tuple negation operator:
  $$Neg\left(s_{i},\alpha \right)=\Delta \left(g-\Delta^{-1}\left(s_{i},\alpha \right)\right)$$

A linguistic term $s_{i},wheres_{i}\in S\equiv \left(s_{i},0\right)$, can be taken as a linguistic 2-tuple by adding to it the value 0 as a symbolic translation. For notation simplicity, this paper sets $\Delta^{-1}\left(s_{i},0\right)=\Delta^{-1}\left(s_{i}\right)$. Therefore, 2-tuple linguistic model can be utilized to represent linguistic preference relations, the linguistic preference relation based on linguistic 2-tuple is formally defined as following:

**Definition** **3.** [5,10]. Let $S=\left\{s_{1},s_{2},\ldots ,s_{g}\right\}$ be the linguistic term set, and let $X=\left\{x_{1},x_{2},\ldots ,x_{n}\right\}\left(n\geq 2\right)$ be a set of alternatives. A linguistic preference relation on a finite set of alternative X is defined as $T^{*}={\left(t_{ij}\right)}_{n\times n}$, where $t_{ij}\in S$, $t_{ii}=s_{\frac{g}{2}}$ and $t_{ji}=Neg\left(t_{ij}\right)$, whose element $t_{ij}$ estimates the preference degree of the alternative $x_{i}$ over $x_{j}$. The following interpretation is assumed: $t_{ij}=s_{\frac{g}{2}}$ indicates indifference between $x_{i}$ and $x_{j}$, $t_{ij}>s_{\frac{g}{2}}$ indicates preference for $x_{i}$ over $x_{j},t_{ij}<s_{\frac{g}{2}}$ indicates preference for $x_{j}$ over $x_{i}$.

### 3.2. Linguistic Preference Relations with Self-Confidence

As we all know, self-confidence of human being has an important influence on decision-making. In order to deal with the self-confidence of decision maker well, Liu et al. [5] present a 2-tuple linguistic preference relations with self-confidence.

Let $S^{SL}=\left\{l_{0},l_{1},l_{2},\ldots ,l_{8}\right\}$ be a linguistic terms set, which allow decision makers to show their self-confidence level over the preference value in a linguistic way. The possible example is shown as following: $S^{SL}=\left\{l_{0}=\mathrm{extremely}\mathrm{poor},l_{1}=\mathrm{very}\mathrm{poor},l_{2}=\mathrm{poor},l_{3}=\mathrm{slightly}\mathrm{poor},l_{4}=\mathrm{fair},l_{5}=\mathrm{slightly}\mathrm{good},l_{6}=\mathrm{good},l_{7}=\mathrm{very}\mathrm{good},l_{8}=\mathrm{extremely}\mathrm{good}\right\}$.

The 2-tuple linguistic preference relation with self-confidence on a finite set of alternatives X = $\left\{x_{1},x_{2},\ldots ,x_{n}\right\}$ is defined as below:

**Definition** **4.** (Liu et al. [5]). *A matrix $T={\left(\left(t_{ij},s_{ij}\right)\right)}_{n\times n}$ is used to describe an ordinal 2-tuple linguistic preference relation with self-confidence on a finite set of alternative X. All elements in $T=\left((t_{ij},s_{ij}\right){)}_{n\times n}$ have two components, the first components $t_{ij}\in S$ representing the ordinal 2-tuple linguistic preference of alternative $x_{i}$ over $x_{j}$, and the second component $s_{ij}\in S^{SL}$ representing the self-confidence level associated to the first part. The following conditions are assumed: $t_{ij}=Neg\left(t_{ji}\right),t_{ii}=s_{\frac{g}{2}},s_{ij}=s_{ji},and\;s_{ii}=l_{g}$.*

**Example** **1.** *Assume T is an SC-LPR that is provided by a decision maker who assesses three alternatives.*

$$T=\left(\begin{array}{ccc} \left(s_{4},l_{8}\right) & \left(s_{5},l_{6}\right) & \left(s_{6},l_{4}\right)\\ \left(s_{3},l_{6}\right) & \left(s_{4},l_{8}\right) & \left(s_{5},l_{4}\right)\\ \left(s_{2},l_{4}\right) & \left(s_{3},l_{4}\right) & \left(s_{4},l_{8}\right) \end{array}\right)$$

In the T, $t_{12}=s_{5}$ means that the preference of the alternative $x_{1}$ over the $x_{2}$ is $s_{5}$, and the $s_{12}=l_{6}$ indicates that the decision maker’s self-confidence level associated to $t_{12}$ is $l_{6}$. The other elements in T can be explained similarly.

### 3.3. Hierarchical Structure in Decision Making

**Figure 1:**
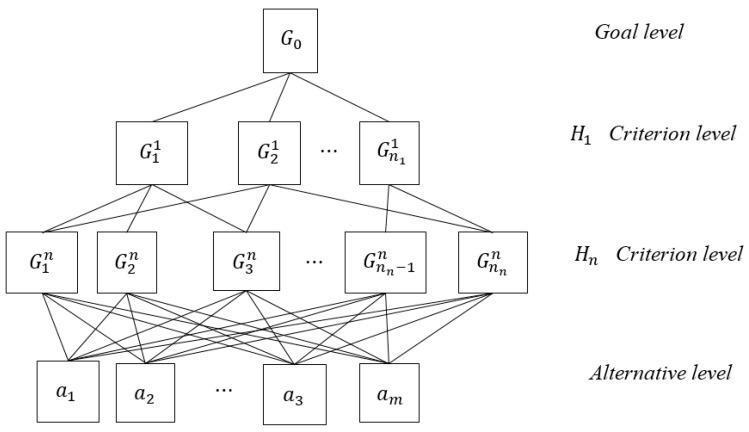
Hierarchical structure.

A hierarchical structure is utilized by decision makers to divide a complicated problem into a hierarchy of more easily comprehended sub-problems [2]. In order to build a linguistic hierarchical model with self-confidence relations, it is necessary to introduce hierarchical structure briefly in this section. Without loss of generality, the following one in Figure 1 is a possible example of hierarchical structure.

Let $H=\left\{H_{k}|k=1,2,\ldots ,n\right\}$ be a hierarchy criteria term set. The term $H_{k}$ in H is the kth level regarding given criteria. Let $n_{k}$ be the number of element in $H_{k}$. Let G be the set of all criteria. Let $A=\left\{a_{1},a_{2},\ldots ,a_{m}\right\}$ be the set of alternatives, which are at the bottom of the hierarchy. There are two groups of criteria in G. The first group consists of the elementary criteria $G_{i}^{n}\left(i=1,2,\ldots ,n_{n}\right)$, which are at the $H_{n}$ criterion level. The other group contains non-elementary criteria, which are at $H_{k}\left(k=1,2,\ldots n-1\right)$ criterion level of the hierarchy. These non-elementary criteria are noted as $G_{i}^{k}\left(k=1,2,\ldots ,n-1;i=1,2,\ldots ,n_{k}\right).$ The set of direct sub-criteria of the $G_{i}^{k}$ is denoted as $E\left(G_{i}^{k}\right)$. $G_{0}$ representing the global objective is the root of the hierarchy.

For example, in Figure 1, $A=\left\{a_{1},a_{2},\ldots ,a_{m}\right\}$ is the set of alternatives. $G_{0}$ is the root of the hierarchy, and the set of all criteria in the hierarchy can be denoted as $G=\left\{G_{1}^{1},G_{2}^{1},\ldots ,G_{n_{n}}^{n}\right\}$. Furthermore, $G_{1}^{1},G_{2}^{1},\ldots ,G_{n_{1}}^{1}$ are non-elementary criteria, while $G_{1}^{n},G_{2}^{n},\ldots ,G_{n_{n}}^{n}$ are elementary criteria. $G_{1}^{n},G_{2}^{n},G_{3}^{n}$ are direct elementary criteria of criterion $G_{1}^{1}$, thus, $E\left(G_{1}^{1}\right)=\left\{G_{1}^{n},G_{2}^{n},G_{3}^{n}\right\}$. Respectively, $E\left(G_{0}\right)=\left\{G_{1}^{1},G_{2}^{1},\ldots ,G_{n_{1}}^{1}\right\}$, $E\left(G_{2}^{1}\right)=\left\{G_{1}^{n},G_{3}^{n},G_{n_{n}}^{n}\right\},$ $E\left(G_{n_{1}}^{1}\right)=\left\{G_{n_{n}-1}^{n},G_{n_{n}}^{n}\right\},$ $E\left(G_{i}^{n}\right)=\left\{a_{1},a_{2},\ldots ,a_{m}\right\}$, where $i=1,2,\ldots ,n_{n}.$

## 4. Linguistic Hierarchy Model with Self-Confidence Preference Relations

In this section, we develop a novel GDM framework: LHM-SCPR. The resolution framework, consistency test regarding SC-LPRs and the nonlinear programming model used in LHM-SCPR are presented.

### 4.1. Framework

**Figure 2:**
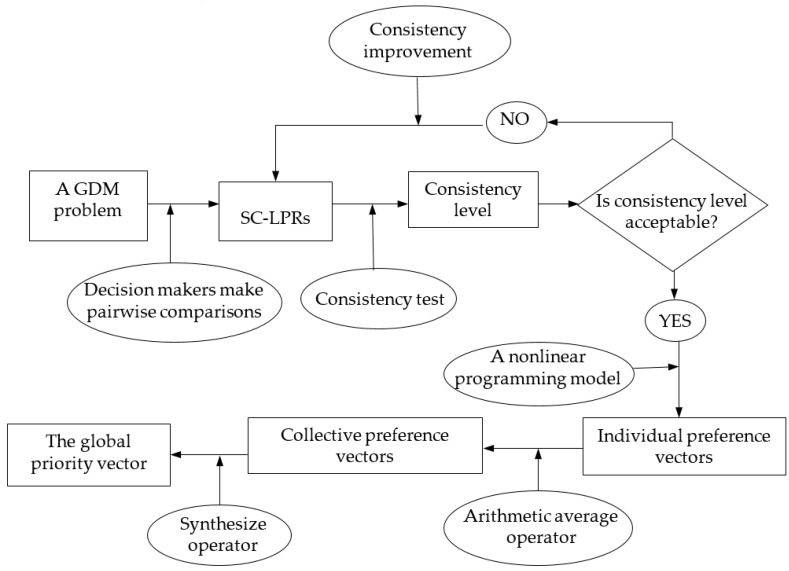
Flowchart of LHM-SCPR.

In this section, a complex GDM solution framework is presented. A group of decision makers shall provide their linguistic preference expression by making pairwise comparison elements in the set $E\left(G_{0}\right)$ and $E\left(G_{i}^{k}\right)$($k=1,2,\ldots ,n;i=1,2,\ldots ,n_{k})$. Hence, LHM-SCPR is utilized to solve this complex GDM problem. According to the common GDM framework (Herrera-Viedam et al. [44]), the flowchart of LHM-SCPR is depicted as Figure 2.

### 4.2. Consistency Test Regarding SC-LPRs

The traditional consistency test is conducted on numerical preference relations (NPRs) with a fixed numerical scale [2], but in LHM-SCPR, consistency test regarding SC-LPR is conducted according to transitive properties.

Up to now, a lot of works regarding the consistency issue of LPR have been conducted [45,46,47]. Clearly, transitivity is an important concept to assess rationality of LPR. Moreover, Liu et al. [5] introduced transitive properties of SC-LPRs to assess rationality of SC-LPRs. Some examples for illustrating consistency test regarding SC-LPRs are presented as following. Let $T={\left(\left(t_{ij},s_{ij}\right)\right)}_{n\times n}$ be SC-LPRs.

*(a) Weak stochastic transitivity at the self-confidence level*$l\in S^{SL}$.

$$t_{ij}\geq s_{\frac{g}{2}},t_{jk}\geq s_{\frac{g}{2}}⟹t_{ik}\geq s_{\frac{g}{2}},\forall i,j,k\;and\;s_{ij}\geq l,\forall i,j.$$

**Example** **2.** *Let $T_{1}={\left(\left(t_{ij},s_{ij}\right)\right)}_{3\times 3}$ be an SC-LPR over alternatives $\left\{x_{1},x_{2},x_{3}\right\}.$*

$$T_{1}=\left(\begin{array}{ccc} \left(s_{4},l_{8}\right) & \left(s_{3},l_{6}\right) & \left(s_{7},l_{4}\right)\\ \left(s_{5},l_{6}\right) & \left(s_{4},l_{8}\right) & \left(s_{6},l_{4}\right)\\ \left(s_{1},l_{4}\right) & \left(s_{2},l_{4}\right) & \left(s_{4},l_{8}\right) \end{array}\right)$$

In $T_{1}$, the preference of the alternative $x_{2}$ over the $x_{1}$, $t_{21}=s_{5}>s_{4}$. The preference of $x_{1}$ is over than $x_{3},$ $t_{13}=s_{7}>s_{4}$, and $x_{2}$ is good than $x_{1}$, $t_{21}=s_{5}>s_{4}$, and $s_{ij}=\left\{l_{g}|l_{g}\geq l_{0},l_{g}\in S^{SL},\forall i,j=1,2,3\right\}$. Thus, $T_{1}$ is an SC-LPR that satisfies weak stochastic transitivity at self-confidence level $l_{0}\in S^{SL},$ The consistency level of $T_{1}$ is considered to be acceptable.

*(b) Strong stochastic transitivity at the self-confidence level*$l\in S^{SL}$.

$$t_{ij}\geq s_{\frac{g}{2}},t_{jk}\geq s_{\frac{g}{2}}⟹t_{ik}\geq max\left(t_{ij},t_{jk}\right),\forall i,j,k\;and\;s_{ij}\geq l,\forall i,j.$$

**Example** **3.** *Let $T_{2}={\left(\left(t_{ij},s_{ij}\right)\right)}_{3\times 3}$ be an SC-LPR over alternatives $\left\{x_{1},x_{2},x_{3}\right\}:$*

$$T_{2}=\left(\begin{array}{ccc} \left(s_{4},l_{8}\right) & \left(s_{5},l_{4}\right) & \left(s_{8},l_{5}\right)\\ \left(s_{3},l_{4}\right) & \left(s_{4},l_{8}\right) & \left(s_{7},l_{3}\right)\\ \left(s_{0},l_{5}\right) & \left(s_{1},l_{3}\right) & \left(s_{4},l_{8}\right) \end{array}\right)$$

In $T_{2}$, the preference of the alternative $x_{1}$ over the $x_{2}$, $t_{12}=s_{5}>s_{4}$. The preference of $x_{2}$ is over than $x_{3}$, $t_{23}=s_{7}>s_{4}$, and $x_{1}$ is good than $x_{3}$, $t_{13}=s_{8}>max\left(t_{12},t_{23}\right)$, and $s_{ij}=\left\{l_{g}|l_{g}\geq l_{0},l_{g}\in S^{SL},\forall i,j=1,2,3\right\}$. Thus, $T_{2}$ is an SC-LPR that satisfies strong stochastic transitivity at self-confidence level $l_{0}\in S^{SL}$, The consistency level of $T_{2}$ is considered to be acceptable.

*(c) Additive transitivity at the self-confidence level* $l\in S^{SL}:$

$$\Delta^{-1}\left(t_{ij}\right)=\Delta^{-1}\left(t_{ik}\right)-\Delta^{-1}\left(t_{jk}\right)+\frac{g}{2}\forall i,j,k\;and\;s_{ij}\geq l,\forall i,j.$$

**Example** **4.** *Let $T_{3}={\left(\left(t_{ij},s_{ij}\right)\right)}_{3\times 3}$ be an SC-LPR over alternatives $\left\{x_{1},x_{2},x_{3}\right\}$:*

$$T_{3}=\left(\begin{array}{ccc} \left(s_{4},l_{8}\right) & \left(s_{5},l_{4}\right) & \left(s_{7},l_{5}\right)\\ \left(s_{3},l_{4}\right) & \left(s_{4},l_{8}\right) & \left(s_{6},l_{6}\right)\\ \left(s_{2},l_{5}\right) & \left(s_{2},l_{6}\right) & \left(s_{4},l_{8}\right) \end{array}\right)$$

In $T_{3}$, the preference of the alternative $x_{1}$ over the $x_{2}$, $t_{12}=s_{5}$. The preference of $x_{2}$ is over than $x_{3}$, $t_{32}=s_{2}$, and $x_{1}$ is over $x_{3}$, $t_{13}=s_{7},so\Delta^{-1}\left(t_{13}\right)=\Delta^{-1}\left(t_{12}\right)-\Delta^{-1}\left(t_{32}\right)+4=5-2+4=7$, and $s_{ij}=\left\{l_{g}|l_{g}\geq l_{0},l_{g}\in S^{SL},\forall i,j=1,2,3\right\}$. Thus, $T_{3}$ is an SC-LPR that satisfies additive transitivity at self-confidence level $l_{0}\in S^{SL}$. The consistency level of $T_{3}$ is considered to be acceptable.

Obviously, the condition of additive transitivity is most restrictive, and the followed by the strong stochastic transitivity and the weak stochastic transitivity. From above information, we know that the consistency level of an SC-LPR satisfied transitive properties is acceptable. In this paper, we assume that the consistency level of an SC-LPR is acceptable when the weak stochastic transitivity of the SC-LPR is satisfied.

### 4.3. Derive Individual Preference Vector from SC-LPRs

In this section, we derive individual preference vector from SC-LPRs by developing a nonlinear programming model with minimum information deviation between decision makers’ preference relations and individual preference vectors.

Firstly, a distance-based optimization model that aims to minimize the error between decision makers’ preference information and their preference vector is proposed, which later is used to develop a nonlinear programming model.

(1) A distance-based optimization model

Let $X=\left\{x_{1},x_{2},\ldots ,x_{n}\right\}$ be a finite set of n alternatives. Let $w={\left(w_{1},w_{2},\ldots ,w_{n}\right)}^{T}$ be a preference vector of a decision maker regarding X, where $\overset{n}{\underset{i=1}{\sum }}w_{i}=1\;and\;w_{i}\geq 0\;for\;\forall i$. Let $T={\left(\left(t_{ij},s_{ij}\right)\right)}_{n\times n}$ be an SC-LPR. According to research of Orlovesky [9], $P={\left(p_{ij}\right)}_{n\times n}$ is an additive preference relation, also called fuzzy preference relations (FPR). The error between the preference value $p_{ij}$ and the preference vector w is $\varepsilon_{ij}$:

(1) $$\varepsilon_{ij}=\frac{1}{2}\left(w_{i}-w_{j}\right)+0.5-p_{ij},i,j=1,2,\ldots ,n$$

Equation (1) denotes the error between the element $p_{ij}$ and $\frac{1}{2}\left(w_{i}-w_{j}\right)+0.5$. In this paper, the linguistic preference values in the SC-LPR are transformed into numerical values using function $\Delta^{-1}\left(t_{ij}\right)/g$ for implementing computation process. Let PT = ${\left(pt_{ij}\right)}_{n\times n}$, where $pt_{ij}=\frac{\Delta^{-1}\left(t_{ij}\right)}{g}.$ Obviously, PT is an additive preference relation. So, similar to Equation (1), we compute the error based on the linguistic preference value $t_{ij}$ and the preference vector w.

Hence, in this paper, the error between the preference value $t_{ij}$ and preference vector w can be similarly defined as:

(2) $$\varepsilon_{ij}=\frac{1}{2}\left(w_{i}-w_{j}\right)+0.5-\frac{\Delta^{-1}\left(t_{ij}\right)}{g},i,j=1,2,\ldots ,n$$

If the individual preference relations are consistent, then it is $\varepsilon_{ij}=0$. The following information deviation is introduced to show the error $\varepsilon_{ij}$ at a self-confidence level of $s_{ij}\left(s_{ij}\in S^{SL}\right)$:

(3) $$z_{ij}=\left|\varepsilon_{ij}*(\Delta^{-1}\left(s_{ij}\right))\right|,i,j=1,2,\ldots ,n$$

The magnification of error $\varepsilon_{ij}$ in Equation (3) is determined by the level of self-confidence $s_{ij}$, the larger value of self-confidence level, the larger magnification will be the error $\varepsilon_{ij}$ regarding the corresponding preference value.

A distance-based optimization model (4) that minimizes the information deviation between decision makers’ preference relations and their individual preference vector is presented. The distance-based optimization model is introduced with p∈[1,∞):

(4) $$d={\left(\overset{n}{\underset{i=1}{\sum }}\overset{n}{\underset{j=1}{\sum }}{\left(z_{ij}\right)}^{p}\right)}^{\frac{1}{p}}$$

The distance-based optimization model (4) is influenced by parameter p: the 1-norm distance (p = 1) is Manhattan distance; the 2-norm distance (p = 2) is the Euclidean distance; the infinity norm distance (p = ∞) is the Chebyshev distance, and Euclidean distance means a straight line distance. In this paper, we use 2-norm distance to obtain the distance-based optimization model. For p=2, the above distance-based optimization model are expressed as Equation (5):

(5) $$d^{*}=\sqrt{\overset{n}{\underset{i=1}{\sum }}\overset{n}{\underset{j=i+1}{\sum }}{\left(z_{ij}\right)}^{2}},i,j=1,2,\ldots ,n;i<j$$

(2) A nonlinear programming model based on the distance-based optimization model 

A nonlinear programming model based on distance-based optimization model is developed to derive individual preference vector from SC-LPRs whose consistency level is acceptable. We set p =2 to minimize information deviation of a decision maker to select a set of optimal preference vectors.

We use four transformed variables in this model: $y_{ij}=\varepsilon_{ij},a_{ij}=\Delta^{-1}\left(t_{ij}\right)$, $b_{ij}=\Delta^{-1}\left(s_{ij}\right)$. The nonlinear programming model is developed as follows:

(6) $$\left\{ \begin{array}{c} minz=\sqrt{\overset{n}{\underset{i=1}{\sum }}\overset{n}{\underset{j=i+1}{\sum }}{\left(z_{ij}\right)}^{2}}\\ s.t.\{\begin{array}{ll} \frac{1}{2}\left(w_{i}-w_{j}\right)+0.5-\frac{a_{ij}}{g}-y_{ij}=0i,j=1,2,\ldots ,n;i<j & \left(a\right)\\ z_{ij}-b_{ij}*y_{ij}\geq 0i,j=1,2,\ldots ,n;i<j & \left(b\right)\\ z_{ij}+b_{ij}*y_{ij}\geq 0i,j=1,2,\ldots ,n;i<j & \left(c\right)\\ w_{1}+w_{2}+w_{3}+\ldots +w_{n}=1 & \left(d\right)\\ w_{i}\geq 0i=1,2,\ldots ,n & \left(e\right)\\ z_{ij}\geq 0i,j=1,2,\ldots ,n;i<j & \left(f\right) \end{array} \end{array}\right.$$

In model (6), the objective is to obtain individual preference vectors $w_{i}\left(i=1,2,\ldots ,n\right)$; constraint (a) means the error between individual preference information and the individual preference vector; constraint (b) and (c) ensure that $z_{ij}-\left|\varepsilon_{ij}*(\Delta^{-1}\left(s_{ij}\right))\right|\geq 0$; constraint (d) ensures the individual preference vector is normalized to sum one; and finally, constraint (e) and (f) guarantee that variables $w_{ij}$ and $z_{ij}$ are nonnegative. The nonlinear programming model is so easy to understand and form that we can use easily available software, such as LINGO, to deal with it.

### 4.4. Detailed Decision Processes for Complex GDM

Based on above information, we develop a novel method, LHM-SCPR, to deal with complex GDM problems. Detailed steps of LHM-SCPR can be generalized as following.

*Step 1.* Build a hierarchical structure model regarding a complex GDM problem, such as Figure 1.

*Step 2.* A group of decision makers provide their SC-LPRs.

Let $D=\left\{d_{c}|c=1,2,\ldots ,h\right\}$ be a term set of decision makers who provide their linguistic preference information by making pairwise comparison elements in the set $E\left(G_{0}\right)$ and $E\left(G_{i}^{k}\right)$($k=1,2,\ldots ,n;i=1,2,\ldots ,n_{k})$. $E\left(G_{0}\right)$ and $E\left(G_{i}^{k}\right)$($k=1,2,\ldots ,n;i=1,2,\ldots ,n_{k})$ can be obtained from the established hierarchical structure in Step1. We denote SC-LPRs as $T_{c}\left(E\left(G_{0}\right)\right)$ and $T_{c}\left(E\left(G_{i}^{k}\right)\right)$, and give an example regarding $T_{c}\left(E\left(G_{0}\right)\right)$:

$$\begin{array}{cc} T_{c}\left(E\left(G_{0}\right)\right) & ={\left(\left(t_{ij,c},s_{ij,c}\right)\right)}_{n_{1}\times n_{1}}\\ & =(\begin{array}{cccc} \left(t_{11,c},s_{11,c}\right) & \left(t_{12,c},s_{12,c}\right) & \ldots & \left(t_{1n_{1},c},s_{1n_{1},c}\right)\\ t_{21,c},s_{21,c} & t_{22,c},s_{22,c} & \ldots & \left(t_{2n_{1},c},s_{2n_{1},c}\right)\\ \vdots & \vdots & \vdots & \vdots \\ \left(t_{n_{1}1,c},s_{n_{1}1,c}\right) & \left(t_{n_{1}2,c},s_{n_{1}2,c}\right) & \ldots & \left(t_{n_{1}n_{1},c},s_{n_{1}n_{1},,c}\right) \end{array}{)}_{n_{1}\times n_{1}} \end{array}$$

*Step 3.* Transitive properties of SC-LPRs are used to assess rationality of every SC-LPRs. decision maker need to modify SC-LPR to improve the consistency, if its consistency level is not acceptable.

*Step 4.* Derive individual preference vectors from SC-LPRs via model (6).

*Step 5.* Individual preference vectors are aggregated to obtain collective preference vector via arithmetic average operator.

We can derive individual preference vectors from each SC-LPRs. Let $W_{C}^{k-1}$ be the individual preference vector of criteria in $H_{k-1}$ criterion level with respect to the global objective. Respectively, the collective preference vector $W^{k-1}$ can be gain as following by aggregating individual preference vectors $W_{C}^{k-1}$:

(7) $$W^{k-1}=\frac{1}{h}\overset{h}{\underset{c=1}{\sum }}W_{C}^{k-1},whereW^{k-1}={\left(w_{1}^{k-1},w_{2}^{k-1},\ldots ,w_{n_{k-1}}^{k-1}\right)}^{T}$$

Let $P_{c,j}^{k}$ be the individual preference vector of criteria in the $H_{k}$ criterion level with respect to the *j*th criteria in the $H_{k-1}$ criterion level, $P_{j}^{k}$ be the collective preference vector regarding $P_{c,j}^{k}$, and $P^{k}$ be the collective preference vector of criteria in $H_{k}$ criterion level with respect to all criteria in the $H_{k-1}$ criterion level:

(8) $$P_{j}^{k}=\frac{1}{h}\sum_{c=1}^{h}P_{c,j}^{k},\mathrm{where}P_{j}^{k}={\left(p_{1j}^{k},p_{2j}^{k},\ldots ,p_{n_{k}j}^{k}\right)}^{T},j=1,2,\ldots ,n_{k-1}$$

(9) $$P^{k}=\left(P_{1}^{k},P_{2}^{k},\ldots ,P_{n_{k-1}}^{k}\right)={\left(p_{ij}^{k}\right)}_{n_{k\times n_{k-1}}}$$

Let $P_{c,j}^{a}$ be the individual preference vector of alternatives in the bottom of the hierarchy with respect to the *j*th criteria in the $H_{n}$ criterion level, $P_{j}^{a}$ be the collective preference vector regarding $P_{c,j}^{a}$, and $P^{a}$ be the collective preference vector of alternatives in the bottom of the hierarchy with respect to all elementary criteria in the $H_{n}$ criterion level:

(10) $$P_{j}^{a}=\frac{1}{h}\sum_{c=1}^{h}P_{c,j}^{a},\mathrm{where}P_{j}^{a}={\left(p_{1j}^{a},p_{2j}^{a},\ldots ,p_{mj}^{a}\right)}^{T},j=1,2,\ldots ,n_{k}$$

(11) $$P^{a}=\left(P_{1}^{a},P_{2}^{a},\ldots ,P_{n_{n}}^{a}\right)={\left(p_{ij}^{a}\right)}_{m\times n_{n}}$$

*Step 6.* Synthesize the collective preference vectors to produce the global priority vector.

We can gain the collective preference vector $W^{k}$ of the criteria in the $H_{k}$ criterion level with respect to the global objective:

(12) $$W^{k}=P^{k}W^{k-1}={\left(w_{1}^{k},w_{2}^{k},\ldots ,w_{n_{k}}^{k}\right)}^{T},\mathrm{where}w_{i}^{k}=\sum_{j=1}^{n_{k-1}}p_{ij}^{k}w_{j}^{k-1},i=1,2,\ldots ,n_{k}$$

By parity of reasoning, the collective preference $W^{n}$ regarding the criteria in the $H_{n}$ criterion level for the global objective can be determined: $W^{n}={\left(w_{1}^{n},w_{2}^{n},\ldots ,w_{n_{n}}^{n}\right)}^{T}$, hence, we can gain the global priority vector $W^{a}$:

(13) $$W^{a}=P^{a}W^{n}={\left(w_{1}^{a},w_{2}^{a},\ldots ,w_{m}^{a}\right)}^{T}\mathrm{where}w_{i}^{a}=\sum_{j=1}^{n_{n}}p_{ij}^{a}w_{j}^{n},i=1,2,\ldots ,m$$

Therefore, we can order the alternatives from the best to the worst according to $W^{a}$.

## 5. Case Study: Co-Regulation of Food Safety

In this section, LHM-SCPR is applied in co-regulation of food safety. In Section 5.1, a complex DM problem regarding co-regulation of food safety is divided into hierarchy simpler sub-problems. In Section 5.2, the details of application are shown. Then, a comparative analysis with a solving process that does not consider multiple self-confidence levels is designed in Section 5.3.

### 5.1. Divide Co-Regulation of Food Safety into Hierarchy Sub-Problems

**Figure 3:**
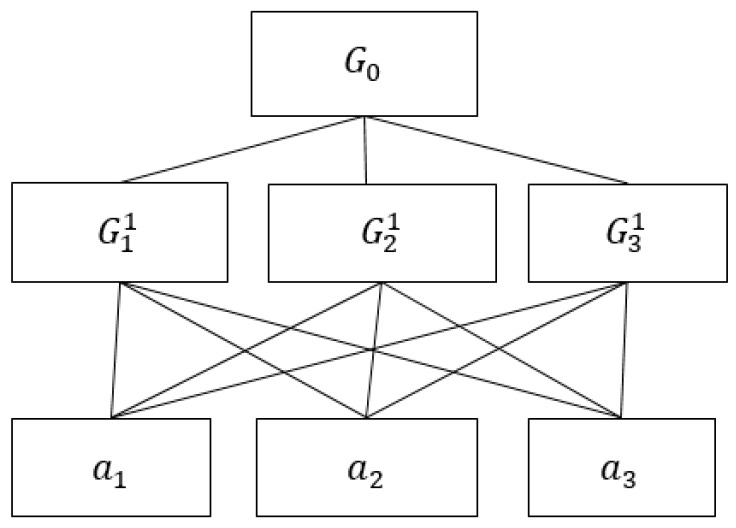
The public participate in co-regulation of food safety.

Public participation can improve co-regualtion effectiveness of public affairs [48]. De Boeck et al. [49] suggested that food safety knowledge has an important impact on individual attitude and behavior regarding food safety. Guo et al. [50] pointed that a lack of social responsibility, inadequate awareness of food risk, pursuit of short-term economic benefit and poor processing technology are root causes of food safety accidents in China. Ergonul [51] and Lim et al. [52] illustrated the importance of consumer awareness and attitude regarding food safety in the food safety management system. On the base of above literatures, participation consciousness, knowledge and skills can be taken as three important criteria. The GDM problem regarding co-regulation of food safety is divided into hierarchy sub-problems in the Figure 3. Elements in Figure 3 are illustrated in Table 2.

(1) Participation consciousness

Participation consciousness of human being has significant influence on individual participation behavior. The public with strong participation consciousness are willing to take part in the public affairs governance. In a word, participation consciousness is the basic premise for the public to participate in co-regulation of food safety.

(2) Participation knowledge

Participation knowledge contains food safety knowledge, legal knowledge and participation knowledge of public affairs. Consumers can identify inedible food with food safety knowledge and defend their right and interest with legal knowledge. Moreover, dealing with food safety problem that is taken as a typical public affairs problem involves much participation knowledge of public affairs.

(3) Participation skills

How to use instruments to identify inedible food and obtain useful information is the basic skill for individual to participate in co-regulation of public affairs. Furthermore, it is essential for the public to master the other participation skill that is cooperating with social organization and governments.

Because the main purpose of this case is to demonstrate the process of this novel method solving a complex GDM problem, we assume that date resources are provided by three decision makers from the Food Safety Supervision Departments, Consumer Association and the Research Institution of Universities. Based on the above three criteria, each decision maker make a pairwise comparison for the provided alternatives, and then provides his/her evaluation.

### 5.2. Application of LHM-SCPR to Co-Regulation of Food Safety

In this section, LHM-SCPR is utilized to analyze the GDM problem regarding the public participation in co-regulation of food safety. Details of application are presented as following.

*Step 1:* A hierarchical structure model regarding co-regulation of food safety is developed in Figure 3.

*Step 2:* Three decision makers provide SC-LPRs.

In Figure 3, the global objective $G_{0}$ is the root of hierarchy, $G_{1}^{1},G_{2}^{1},G_{3}^{1}$ are elementary criteria, and $a_{1},a_{2},a_{3}$ are alternatives at the bottom of the hierarchy. Moreover, $G_{1}^{1},G_{2}^{1},G_{3}^{1}$ are direct sub-criteria of global objective $G_{0}$, thus, $E\left(G_{0}\right)=\left\{G_{1}^{1},G_{2}^{1},G_{3}^{1}\right\}$, respectively, $E\left(G_{1}^{1}\right)=\left\{a_{1},a_{2},a_{3}\right\}$, $E\left(G_{2}^{1}\right)=\left\{a_{1},a_{2},a_{3}\right\}$, $E\left(G_{3}^{1}\right)=\left\{a_{1},a_{2},a_{3}\right\}$. Decision makers $d_{c}\left(c=1,2,3\right)$ express their SC-LPRs by making pairwise comparison elements in the above sets: $E\left(G_{0}\right),E\left(G_{1}^{1}\right),E\left(G_{2}^{1}\right),E\left(G_{2}^{1}\right)$, all SC-LPRs can be obtained as follows:

$$T_{1}\left(E\left(G_{0}\right)\right)=\left(\begin{array}{ccc} \left(s_{4},l_{8}\right) & \left(s_{5},l_{1}\right) & \left(s_{6},l_{4}\right)\\ \left(s_{3},l_{1}\right) & \left(s_{4},l_{8}\right) & \left(s_{3},l_{7}\right)\\ \left(s_{2},l_{4}\right) & \left(s_{5},l_{7}\right) & \left(s_{4},l_{8}\right) \end{array}\right),T_{2}\left(E\left(G_{0}\right)\right)=\left(\begin{array}{ccc} \left(s_{4},l_{8}\right) & \left(s_{4},l_{1}\right) & \left(s_{6},l_{2}\right)\\ \left(s_{4},l_{1}\right) & \left(s_{4},l_{8}\right) & \left(s_{5},l_{6}\right)\\ \left(s_{2},l_{2}\right) & \left(s_{3},l_{6}\right) & \left(s_{4},l_{8}\right) \end{array}\right)$$

$$T_{3}\left(E\left(G_{0}\right)\right)=\left(\begin{array}{ccc} \left(s_{4},l_{8}\right) & \left(s_{5},l_{6}\right) & \left(s_{4},l_{4}\right)\\ \left(s_{3},l_{6}\right) & \left(s_{4},l_{8}\right) & \left(s_{2},l_{4}\right)\\ \left(s_{4},l_{4}\right) & \left(s_{6},l_{4}\right) & \left(s_{4},l_{8}\right) \end{array}\right),T_{1}\left(E\left(G_{1}^{1}\right)\right)=\left(\begin{array}{ccc} \left(s_{4},l_{8}\right) & \left(s_{5},l_{2}\right) & \left(s_{6},l_{5}\right)\\ \left(s_{3},l_{2}\right) & \left(s_{4},l_{8}\right) & \left(s_{4},l_{8}\right)\\ \left(s_{2},l_{5}\right) & \left(s_{4},l_{8}\right) & \left(s_{4},l_{8}\right) \end{array}\right)$$

$$T_{2}\left(E\left(G_{1}^{1}\right)\right)=\left(\begin{array}{ccc} \left(s_{4},l_{8}\right) & \left(s_{5},l_{1}\right) & \left(s_{7},l_{3}\right)\\ \left(s_{3},l_{1}\right) & \left(s_{4},l_{8}\right) & \left(s_{4},l_{6}\right)\\ \left(s_{1},l_{3}\right) & \left(s_{4},l_{6}\right) & \left(s_{4},l_{8}\right) \end{array}\right),T_{3}\left(E\left(G_{1}^{1}\right)\right)=\left(\begin{array}{ccc} \left(s_{4},l_{8}\right) & \left(s_{5},l_{2}\right) & \left(s_{6},l_{6}\right)\\ \left(s_{3},l_{2}\right) & \left(s_{4},l_{8}\right) & \left(s_{3},l_{7}\right)\\ \left(s_{2},l_{6}\right) & \left(s_{5},l_{7}\right) & \left(s_{4},l_{8}\right) \end{array}\right)$$

$$T_{1}\left(E\left(G_{2}^{1}\right)\right)=\left(\begin{array}{ccc} \left(s_{4},l_{8}\right) & \left(s_{1},l_{4}\right) & \left(s_{5},l_{5}\right)\\ \left(s_{7},l_{4}\right) & \left(s_{4},l_{8}\right) & \left(s_{5},l_{7}\right)\\ \left(s_{3},l_{5}\right) & \left(s_{3},l_{7}\right) & \left(s_{4},l_{8}\right) \end{array}\right)T_{2}\left(E\left(G_{2}^{1}\right)\right)=\left(\begin{array}{ccc} \left(s_{4},l_{8}\right) & \left(s_{4},l_{3}\right) & \left(s_{6},l_{6}\right)\\ \left(s_{4},l_{3}\right) & \left(s_{4},l_{8}\right) & \left(s_{5},l_{3}\right)\\ \left(s_{2},l_{6}\right) & \left(s_{3},l_{3}\right) & \left(s_{4},l_{8}\right) \end{array}\right)$$

$$T_{3}\left(E\left(G_{2}^{1}\right)\right)=\left(\begin{array}{ccc} \left(s_{4},l_{8}\right) & \left(s_{3},l_{2}\right) & \left(s_{5},l_{6}\right)\\ \left(s_{5},l_{2}\right) & \left(s_{4},l_{8}\right) & \left(s_{5},l_{4}\right)\\ \left(s_{3},l_{6}\right) & \left(s_{3},l_{4}\right) & \left(s_{4},l_{8}\right) \end{array}\right),T_{1}\left(E\left(G_{3}^{1}\right)\right)=\left(\begin{array}{ccc} \left(s_{4},l_{8}\right) & \left(s_{5},l_{7}\right) & \left(s_{3},l_{2}\right)\\ \left(s_{3},l_{7}\right) & \left(s_{4},l_{8}\right) & \left(s_{1},l_{6}\right)\\ \left(s_{5},l_{2}\right) & \left(s_{7},l_{6}\right) & \left(s_{4},l_{8}\right) \end{array}\right)$$

$$T_{2}\left(E\left(G_{3}^{1}\right)\right)=\left(\begin{array}{ccc} \left(s_{4},l_{8}\right) & \left(s_{4},l_{7}\right) & \left(s_{3},l_{3}\right)\\ \left(s_{4},l_{7}\right) & \left(s_{4},l_{8}\right) & \left(s_{1},l_{6}\right)\\ \left(s_{5},l_{3}\right) & \left(s_{7},l_{6}\right) & \left(s_{4},l_{8}\right) \end{array}\right),T_{3}\left(E\left(G_{3}^{1}\right)\right)=\left(\begin{array}{ccc} \left(s_{4},l_{8}\right) & \left(s_{5},l_{5}\right) & \left(s_{1},l_{4}\right)\\ \left(s_{3},l_{5}\right) & \left(s_{4},l_{8}\right) & \left(s_{2},l_{7}\right)\\ \left(s_{7},l_{4}\right) & \left(s_{6},l_{7}\right) & \left(s_{4},l_{8}\right) \end{array}\right)$$

*Step 3:* Assess rationality of all SC-LPRs.

In $T_{1}\left(E\left(G_{0}\right)\right)$, the preference of the alternative $x_{1}$ over the $x_{3}$, $t_{13}=s_{6}>s_{4}$. The preference of $x_{3}$ is over than $x_{2}$, $t_{32}=s_{5}>s_{4}$, and $x_{1}$ is good than $x_{2}$, $t_{12}=s_{5}>s_{4}$, and $s_{ij}=\left\{l_{g}|l_{g}\geq l_{0},l_{g}\in S^{SL},\forall i,j=1,2,3\right\}$. Thus, $T_{1}\left(E\left(G_{0}\right)\right)$ satisfies the weak stochastic transitivity at self-confidence level $l_{0}\in S^{SL}$. It is considered to be acceptable consistent.

Obviously:$\left(E\left(G_{0}\right)\right),T_{3}\left(E\left(G_{0}\right)\right),T_{1}\left(E\left(G_{1}^{1}\right)\right),T_{2}\left(E\left(G_{1}^{1}\right)\right),T_{3}\left(E\left(G_{1}^{1}\right)\right),T_{1}\left(E\left(G_{2}^{1}\right)\right),T_{2}\left(E\left(G_{2}^{1}\right)\right),T_{3}\left(E\left(G_{2}^{1}\right)\right),T_{1}\left(E\left(G_{3}^{1}\right)\right),T_{2}\left(E\left(G_{3}^{1}\right)\right),T_{3}\left(E\left(G_{3}^{1}\right)\right)$ satisfy the weak stochastic transitivity at self-confidence level $l_{0}\in S^{SL}$.

*Step 4:* Derive individual preference vectors from SC-LPRs via model (6).

Compute the individual preference vector regarding these above SC-LPRs by model (6), we obtain three decision makers’ individual preference vector of the above SC-LPRs. The detailed results are shown in Table 3.

*Step 5:* Obtain collective preference vector by aggregating individual preference vectors.

$W_{1}^{1},W_{2}^{1},W_{3}^{1}$ are individual preference vectors of criteria in the criterion level with respect to the global objective $G_{0}$. We can obtain the collective preference vector $W^{1}$ by Equation (7):

$$W^{1}=\frac{1}{3}\left(W_{1}^{1}+W_{2}^{1}+W_{3}^{1}\right)={\left(0.590,0.153,0.257\right)}^{T}$$

$P_{1,1}^{a},P_{2,1}^{a},P_{3,1}^{a}$ are individual preference vectors of alternatives in the bottom of hierarchy with respect to the first criterion $G_{1}^{1}$ in criterion level. The collective preference vector $P_{1}^{a}$ is calculated by Equation (10).

$$P_{1}^{a}=\frac{1}{3}\left(P_{1,1}^{a}+P_{2,1}^{a}+P_{3,1}^{a}\right)={\left(0.742,0.097,0.164\right)}^{T}$$

$P_{1,2}^{a},P_{2,2}^{a},P_{3,2}^{a}$ are individual preference vectors of alternatives in the bottom of hierarchy with respect to the second criterion $G_{2}^{1}$ in criterion level. The collective preference vector $P_{2}^{a}$ is calculated by Equation (10).

$$P_{1}^{a}=\frac{1}{3}\left(P_{1,2}^{a}+P_{2,2}^{a}+P_{3,2}^{a}\right)={\left(0.432,0469,0.099\right)}^{T}$$

$P_{1,3}^{a},P_{2,3}^{a},P_{3,3}^{a}$ are individual preference vectors of alternatives in the bottom of hierarchy with respect to the third criterion $G_{3}^{1}$ in criterion level. We can calculate the collective preference vector $P_{3}^{a}$ by Equation (10).

$$P_{3}^{a}=\frac{1}{3}\left(P_{1,3}^{a}+P_{2,3}^{a}+P_{3,3}^{a}\right)={\left(0.341,0.068,0.591\right)}^{T}$$

Hence, $P^{a}$, the collective preference vector of alternatives in the bottom of the hierarchy with respect to all criteria in the criterion level, can be obtained by Equation (11).

$$P^{a}=\left(P_{1}^{a},P_{2}^{a},P_{3}^{a}\right)=\left(\begin{array}{ccc} 0.742 & 0.432 & 0.341\\ 0.097 & 0.469 & 0.068\\ 0.164 & 0.099 & 0.591 \end{array}\right)$$

*Step 6:* Synthesize these collective preference vectors to produce the global priority vector.

The global priority vector $W^{a}$ can be calculated by Equation (14):

$$W^{a}=P^{a}*W^{1}=\left(\begin{array}{ccc} 0.742 & 0.432 & 0.341\\ 0.097 & 0.469 & 0.068\\ 0.164 & 0.099 & 0.591 \end{array}\right)*\left(\begin{array}{c} 0.590\\ 0.153\\ 0.257 \end{array}\right)={\left(0.591,0.146,0.263\right)}^{T}$$

Thus, the ranking of alternatives is $a_{1}≻a_{3}≻a_{2}$. So, the optimal measure is $a_{1}$, and the followed by the $a_{3}$ and $a_{2}.$

### 5.3. Comparative Analysis

In order to further verify the validity of proposed LHM-SCPR in this paper, a comparative analysis is given in this section.

As we know, decision makers express their preference information with LPRs, in which their self-confidence levels regarding all linguistic preference values are the same, that is $l_{ij}=s_{8}\in S^{SL}.$ Thus, LPRs can be taken as a special case of SC-LPRs. We make a comparative analysis by changing the self-confidence level regarding these SC-LPRs provided in Section 5.2. SC-LPRs with same self-confidence level are presented as following:

$$T_{1}^{\prime }\left(E\left(G_{0}\right)\right)=\left(\begin{array}{ccc} \left(s_{4},l_{8}\right) & \left(s_{5},l_{8}\right) & \left(s_{6},l_{8}\right)\\ \left(s_{3},l_{8}\right) & \left(s_{4},l_{8}\right) & \left(s_{3},l_{8}\right)\\ \left(s_{2},l_{8}\right) & \left(s_{5},l_{8}\right) & \left(s_{4},l_{8}\right) \end{array}\right),T_{2}^{\prime }\left(E\left(G_{0}\right)\right)=\left(\begin{array}{ccc} \left(s_{4},l_{8}\right) & \left(s_{4},l_{8}\right) & \left(s_{6},l_{8}\right)\\ \left(s_{4},l_{8}\right) & \left(s_{4},l_{8}\right) & \left(s_{5},l_{8}\right)\\ \left(s_{2},l_{8}\right) & \left(s_{3},l_{8}\right) & \left(s_{4},l_{8}\right) \end{array}\right)$$

$$T_{3}^{\prime }\left(E\left(G_{0}\right)\right)=\left(\begin{array}{ccc} \left(s_{4},l_{8}\right) & \left(s_{5},l_{8}\right) & \left(s_{4},l_{8}\right)\\ \left(s_{3},l_{8}\right) & \left(s_{4},l_{8}\right) & \left(s_{2},l_{8}\right)\\ \left(s_{4},l_{8}\right) & \left(s_{6},l_{8}\right) & \left(s_{4},l_{8}\right) \end{array}\right),T_{1}^{\prime }\left(E\left(G_{1}^{1}\right)\right)=\left(\begin{array}{ccc} \left(s_{4},l_{8}\right) & \left(s_{5},l_{8}\right) & \left(s_{6},l_{8}\right)\\ \left(s_{3},l_{8}\right) & \left(s_{4},l_{8}\right) & \left(s_{4},l_{8}\right)\\ \left(s_{2},l_{8}\right) & \left(s_{4},l_{8}\right) & \left(s_{4},l_{8}\right) \end{array}\right)$$

$$T_{2}^{\prime }\left(E\left(G_{1}^{1}\right)\right)=\left(\begin{array}{ccc} \left(s_{4},l_{8}\right) & \left(s_{5},l_{8}\right) & \left(s_{7},l_{8}\right)\\ \left(s_{3},l_{8}\right) & \left(s_{4},l_{8}\right) & \left(s_{4},l_{8}\right)\\ \left(s_{1},l_{8}\right) & \left(s_{4},l_{8}\right) & \left(s_{4},l_{8}\right) \end{array}\right),T_{3}^{\prime }\left(E\left(G_{1}^{1}\right)\right)=\left(\begin{array}{ccc} \left(s_{4},l_{8}\right) & \left(s_{5},l_{8}\right) & \left(s_{6},l_{8}\right)\\ \left(s_{3},l_{8}\right) & \left(s_{4},l_{8}\right) & \left(s_{3},l_{8}\right)\\ \left(s_{2},l_{8}\right) & \left(s_{5},l_{8}\right) & \left(s_{4},l_{8}\right) \end{array}\right)$$

$$T_{1}^{\prime }\left(E\left(G_{2}^{1}\right)\right)=\left(\begin{array}{ccc} \left(s_{4},l_{8}\right) & \left(s_{1},l_{8}\right) & \left(s_{5},l_{8}\right)\\ \left(s_{7},l_{8}\right) & \left(s_{4},l_{8}\right) & \left(s_{5},l_{8}\right)\\ \left(s_{3},l_{8}\right) & \left(s_{3},l_{8}\right) & \left(s_{4},l_{8}\right) \end{array}\right),T_{2}^{\prime }\left(E\left(G_{2}^{1}\right)\right)=\left(\begin{array}{ccc} \left(s_{4},l_{8}\right) & \left(s_{4},l_{8}\right) & \left(s_{6},l_{8}\right)\\ \left(s_{4},l_{8}\right) & \left(s_{4},l_{8}\right) & \left(s_{5},l_{8}\right)\\ \left(s_{2},l_{8}\right) & \left(s_{3},l_{8}\right) & \left(s_{4},l_{8}\right) \end{array}\right)$$

$$T_{3}^{\prime }\left(E\left(G_{2}^{1}\right)\right)=\left(\begin{array}{ccc} \left(s_{4},l_{8}\right) & \left(s_{3},l_{8}\right) & \left(s_{5},l_{8}\right)\\ \left(s_{5},l_{8}\right) & \left(s_{4},l_{8}\right) & \left(s_{5},l_{8}\right)\\ \left(s_{3},l_{8}\right) & \left(s_{3},l_{8}\right) & \left(s_{4},l_{8}\right) \end{array}\right),T_{1}^{\prime }\left(E\left(G_{3}^{1}\right)\right)=\left(\begin{array}{ccc} \left(s_{4},l_{8}\right) & \left(s_{5},l_{8}\right) & \left(s_{3},l_{8}\right)\\ \left(s_{3},l_{8}\right) & \left(s_{4},l_{8}\right) & \left(s_{1},l_{8}\right)\\ \left(s_{5},l_{8}\right) & \left(s_{7},l_{8}\right) & \left(s_{4},l_{8}\right) \end{array}\right)$$

$$T_{2}^{\prime }\left(E\left(G_{3}^{1}\right)\right)=\left(\begin{array}{ccc} \left(s_{4},l_{8}\right) & \left(s_{4},l_{8}\right) & \left(s_{3},l_{8}\right)\\ \left(s_{4},l_{8}\right) & \left(s_{4},l_{8}\right) & \left(s_{1},l_{8}\right)\\ \left(s_{5},l_{8}\right) & \left(s_{7},l_{8}\right) & \left(s_{4},l_{8}\right) \end{array}\right),T_{3}^{\prime }\left(E\left(G_{3}^{1}\right)\right)=\left(\begin{array}{ccc} \left(s_{4},l_{8}\right) & \left(s_{5},l_{5}\right) & \left(s_{1},l_{4}\right)\\ \left(s_{3},l_{5}\right) & \left(s_{4},l_{8}\right) & \left(s_{2},l_{7}\right)\\ \left(s_{7},l_{4}\right) & \left(s_{6},l_{7}\right) & \left(s_{4},l_{8}\right) \end{array}\right)$$

Obviously, the above SC-LPRs all satisfy the weak stochastic transitivity at the self-confidence level $l_{0}.$ Then, we carry on the following calculations according to the procedure of LHM-SCPR and omit the specific calculation. Results of preference vector regarding the above SC-LPRs are shown as Table 4.

So, the collective preference vector ${P^{a}}^{\prime }$ regarding alternatives in the bottom of the hierarchy with respect to all criteria can be obtained:

$${P^{a}}^{\prime }=\left({P_{1}^{a}}^{\prime },{P_{2}^{a}}^{\prime },{P_{3}^{a}}^{\prime }\right)=\left(\begin{array}{ccc} 0.694 & 0.379 & 0.425\\ 0.178 & 0.552 & 0.073\\ 0.127 & 0.068 & 0.502 \end{array}\right)$$

so the global priority vector ${W^{a}}^{\prime }$ can be obtained as following:

$${W^{a}}^{\prime }={P^{a}}^{\prime }*{W^{1}}^{\prime }=\left(\begin{array}{ccc} 0.694 & 0.379 & 0.425\\ 0.178 & 0.552 & 0.073\\ 0.127 & 0.068 & 0.502 \end{array}\right)*\left(\begin{array}{c} 0.555\\ 0.221\\ 0.222 \end{array}\right)={\left(0.563,0.236,0.198\right)}^{T}$$

Thus, the ranking of alternatives is $a_{1}≻a_{2}≻a_{3},$ we can know from the result that the ranking of alternatives in Section 5.3 is different from the result which we have obtained in Section 5.2. Thus, we can know that multiple self-confidence levels have an important influence on the final decision in GDM problems.

Doubtlessly, the alternative $a_{1}$ is the optimal measure that is possibly to enhance individual participation consciousness and enrich their participation knowledge. The alternative $a_{3}$ can help individual to form a participation habit and strengthen his /her participation consciousness. For example, with the development of internet, consumer can expose food safety accidents on the internet. Then the government will deal with this kind of food safety issue under the pressure from the public, and the food enterprises who are responsible for the food safety accident may be at the risk of failure. Moreover, at present, exposing food safety on the internet is the main method for the public to participate in the co-regulation of food safety. So, more convenient and effective methods of participation need to be proposed and be applied. The result in Section 5.3 illustrate that the alternative $a_{2}$ is more important than $a_{3}$. However, according to experiences of foreign countries, it seems that application of methods and procedures of public affairs governance is necessary for better results. As we all know, food safety knowledge is so complex that it is difficult for the public to master all. Thus, when we analyze co-regulation of food safety by the use of LHM-SCPR, we can make a more reasonable decision.

Based on the comparative analysis, it is not difficult to find that different results can be gained by analysing a problem from different perspectives. In real-world situations, a DM problem is likely to involve several stakeholders, whose experience and knowledge are different. Therefore, discussing a problem from perspective of GDM is beneficial for us to make a reasonable decision. Moreover, this proposal can also be applied in addressing other GDM problems, such as risk assessment and performance evaluation, and so on. If a complex GDM problem can be divide into hierarchy simpler sub-problems that can’t be expressed with exact number value, this proposed method can be applied in it.

## 6. Discussion: Contributions and Limitations

In this section, we present some contributions and limitations of our proposal.

### 6.1. Contributions

In the following, we summarize the main contributions of our paper from two aspects:

(1) This paper proposed a novel GDM method for complex DM problems by constructing a model based on the linguistic preference relations with self-confidence and hierarchical structure.

The existing GDM method, such as traditional AHP and extended AHP, are describe by multiplicative preference relations and fuzzy preference relations. Dong et al. [10] recently proposed linguistic preference relations, which have shown its richness and convenience in expressing decision makers’ preference information. However, the existing AHP did not consider a non-negligible problem that individual preference information regarding an alternative is often vague and can’t be expressed with a crisp number. The proposal LHM-SCPR combine hierarchical structure in AHP and self-confidence linguistic preference relations, analysing complicated GDM problems. In the following, we compare the proposed LHM-SCPR with some existing GDM methods to show the advantage of our proposal.

Comparison with [26,27,28,29,30,38,39,40]. The individual preference information is expressed with multiplicative preference relations and fuzzy preference relations in [26,27,28,29,30,38,39,40]. Compared with these studies, we adopted a more flexible linguistic preference expression, which can allow decision makers to express their multiple self-confidence levels when providing their linguistic preference relations. 

Comparison with [2,53,54]. The priority vectors are derived with prioritization method in [53,54] and Eigenvalue methods in [2]. Compared with these studies, we derive preference vectors from SC-LPRs by constructing a programming model based on the distance-based optimization model, which minimizes information deviation between decision makers’ preference relations and individual preference vectors.

(2) This paper that analyses food safety governance in China from the GDM perspective enrich the method of discussing food safety governance in China.

Comparison with [49,50,51,52]. The structural equation model has been used to analyse food safety problems in [49,50,51,52]. Compared with these studies, we discussed the food safety governance from the perspective of GDM.

### 6.2. Limitations

There are some limitations of the novel method, and these limitations need to be dealt with in the future research.

(1) Consensus is one of important aspect within an interactive group [31,32,55,56,57,58,59], it has been widely analysed in some researches so as to guide decision makers to reach a consensus before making a decision, so that the obtained solution is acceptable for group. However, in this paper, we just only taken the consistency of SC-LPRs into consideration. It is one of the main limitations that we didn’t discussed consensus in the novel method.
(2) Real-world GDM problems involve not only mathematical aspects but also psychological behaviors, such as self-confidence and personalized individual semantics [60]. Because different words mean different things to different people, we argue that it will be interesting to investigate the personalized individual semantics of decision makers in GDM problems.

## 7. Conclusions

In GDM, self-confidence is an important factor that cannot be ignored due to its influence on the final decision making results, and hierarchical structure is widely used to divide complicated GDM problems. In order to improve the quality of decision making, this paper design an novel method for handling complex GDM problems, and apply it to the co-regulation of food safety. The main works of this paper are summarized below:

(1) We design a novel GDM framework, called LHM-SCPR, which connects hierarchical structure and self-confidence linguistic preference relations for complicated GDM problems.
(2) We build a nonlinear programming model with minimum information deviation between decision makers’ preference relations and their individual preference vectors to gain individual preference vectors from SC-LPRs.
(3) To illustrate the application value of the proposed LHM-SCPR, it is applied to analyse food safety governance in order to improve governance effectiveness.

With the rapid development of science and technology, some activities are more complicated and involve more and more decision makers, such as social networks [61], water management [62], and environment pollution management [63] This implies large scale group decision making (LSGDM) has become a research hotspot [64,65,66]. Hence, we argue that it will be interesting in future to investigate LHM-SCPR under LSGDM context.

## Figures and Tables

**Table 1:** Major food safety accidents in recent years in China.

Year	Major Food Safety Accidents
2005	“Malachite Green Seafood”, “Leather Milk
2006	“Sudanese Red Duck Egg”
2008	“Melamine Milk Powder”
2009	“Leather Milk”
2010	“Waste Cooking Oil”
2011	“Leather Milk”, “Poisonous Pork with Clenbuterol”
2013	“Cadmium Rice”, “Poisonous Ginger”
2015	“Expired Frozen Meat”
2016	“Takeaway Hygiene”
2018	“Tongrentang Honey”, “Takeaway Hygiene”

**Table 2:** Description of elements in Figure 3.

	Element	Semantics
Goal	$G_{0}$	Public participate in co-regulation of food safety
Criterion	$G_{1}^{1}$	Participation consciousness
	$G_{2}^{1}$	participation knowledge
	$G_{3}^{1}$	Participation skills
Alternative	$a_{1}$	Popularize legal knowledge and strengthen the legal system
	$a_{2}$	Popularize food safety knowledge
	$a_{3}$	Popularize methods and procedures of public affairs governance

**Table 3:** Results of decision makers’ individual preference vector.

SC-PRLs	z	Individual Preference Vector
$T_{1}\left(E\left(G_{0}\right)\right)$	0.247	$W_{1}^{1}={\left(0.747,0.006,0.247\right)}^{T}$
$T_{2}\left(E\left(G_{0}\right)\right)$	0.123	$W_{2}^{1}={\left(0.581,0.337,0.081\right)}^{T}$
$T_{3}\left(E\left(G_{0}\right)\right)$	0.416	$W_{3}^{1}={\left(0.442,0.115,0.442\right)}^{T}$
$T_{1}\left(E\left(G_{1}^{1}\right)\right)$	0.243	$P_{1,1}^{a}={\left(0.662,0.176,0.176\right)}^{T}$
$T_{2}\left(E\left(G_{1}^{1}\right)\right)$	0.246	$P_{2,1}^{a}={\left(0.828,0.092,0.079\right)}^{T}$
$T_{3}\left(E\left(G_{1}^{1}\right)\right)$	0.480	$P_{3,1}^{a}={\left(0.737,0.025,0.237\right)}^{T}$
$T_{1}\left(E\left(G_{2}^{1}\right)\right)$	1.302	$P_{1,2}^{a}={\left(0.355,0.539,0.105\right)}^{T}$
$T_{2}\left(E\left(G_{2}^{1}\right)\right)$	0.265	$P_{2,2}^{a}={\left(0.542,0.417,0.042\right)}^{T}$
$T_{3}\left(E\left(G_{2}^{1}\right)\right)$	0.224	$P_{3,2}^{a}={\left(0.400,0.450,0.042\right)}^{T}$
$T_{1}\left(E\left(G_{3}^{1}\right)\right)$	0.569	$P_{1,3}^{a}={\left(0.368,0.013,0.618\right)}^{T}$
$T_{2}\left(E\left(G_{3}^{1}\right)\right)$	1.138	$P_{2,3}^{a}={\left(0.321,0.109,0.571\right)}^{T}$
$T_{3}\left(E\left(G_{3}^{1}\right)\right)$	1.000	$P_{3,3}^{a}={\left(0.333,0.083,0.583\right)}^{T}$

**Table 4:** Results of preference vector.

SC-PRLs	z	Individual Preference Vector	Collective Preference Vector
$T_{1}^{\prime }\left(E\left(G_{0}\right)\right)$	1.412	${W_{1}^{1}}^{\prime }={\left(0.667,0.166,0.166\right)}^{T}$	${W^{1}}^{\prime }={\left(0.555,0.221,0.222\right)}^{T}$
$T_{2}^{\prime }\left(E\left(G_{0}\right)\right)$	0.700	${W_{2}^{1}}^{\prime }={\left(0.542,0.416,0.042\right)}^{T}$	
$T_{3}^{\prime }\left(E\left(G_{0}\right)\right)$	0.707	${W_{3}^{1}}^{\prime }={\left(0.458,0.083,0.458\right)}^{T}$	
$T_{1}^{\prime }\left(E\left(G_{1}^{1}\right)\right)$	0.707	${P_{1,1}^{a}}^{\prime }={\left(0.625,0.250,0.125\right)}^{T}$	${P_{1}^{a}}^{\prime }={\left(0.694,0.178,0.127\right)}^{T}$
$T_{2}^{\prime }\left(E\left(G_{1}^{1}\right)\right)$	1.414	${P_{2,1}^{2,1}}^{\prime }={\left(0.750,0.250,0.000\right)}^{T}$	
$T_{3}^{\prime }\left(E\left(G_{1}^{1}\right)\right)$	1.410	${P_{3,1}^{2,1}}^{\prime }={\left(0.667,0.167,0.167\right)}^{T}$	
$T_{1}^{\prime }\left(E\left(G_{2}^{1}\right)\right)$	2.121	${P_{1,2}^{a}}^{\prime }={\left(0.292,0.667,0.04\right)}^{T}$	${P_{2}^{a}}^{\prime }={\left(0.379,0.552,0.068\right)}^{T}$
$T_{2}^{\prime }\left(E\left(G_{2}^{1}\right)\right)$	0.707	${P_{2,2}^{a}}^{\prime }={\left(0.471,0.490,0.039\right)}^{T}$	
$T_{3}^{\prime }\left(E\left(G_{2}^{1}\right)\right)$	0.707	${P_{3,2}^{a}}^{\prime }={\left(0.375,0.500,0.125\right)}^{T}$	
$T_{1}^{\prime }\left(E\left(G_{3}^{1}\right)\right)$	0.707	${P_{1,3}^{a}}^{\prime }={\left(0.375,0.000,0.618\right)}^{T}$	${P_{3}^{a}}^{\prime }={\left(0.425,0.073,0.502\right)}^{T}$
$T_{2}^{\prime }\left(E\left(G_{3}^{1}\right)\right)$	1.414	${P_{2,3}^{a}}^{\prime }={\left(0.333,0.083,0.583\right)}^{T}$	
$T_{3}^{\prime }\left(E\left(G_{3}^{1}\right)\right)$	2.000	${P_{3,3}^{a}}^{\prime }={\left(0.333,0.083,0.583\right)}^{T}$	
